# Wetlands are keystone habitats for jaguars in an intercontinental biodiversity hotspot

**DOI:** 10.1371/journal.pone.0221705

**Published:** 2019-09-11

**Authors:** Joe J. Figel, Sebastián Botero-Cañola, German Forero-Medina, Juan David Sánchez-Londoño, Leonor Valenzuela, Reed F. Noss

**Affiliations:** 1 Department of Biology, University of Central Florida, Orlando, Florida, United States of America; 2 Harold W Manter Laboratory of Parasitology, University of Nebraska State Museum and School of Biological Sciences, University of Nebraska, Lincoln, Nebraska, United States of America; 3 Instituto de Biología, Grupo de Mastozoología, Universidad de Antioquia, Medellín, Colombia; 4 Wildlife Conservation Society-Colombia Program, Cali, Colombia; 5 Facultad de Ciencias y Biotecnología, Universidad CES, Medellín, Colombia; 6 Fundación BioDiversa, Bogotá, Colombia; 7 Florida Institute for Conservation Science, Chuluota, Florida, United States of America; Panthera, UNITED STATES

## Abstract

Agricultural development was the major contributor to South America’s designation as the continent with the highest rates of forest loss from 2000–2012. As the apex predator in the Neotropics, jaguars (*Panthera onca*) are dependent on forest cover but the species’ response to habitat fragmentation in heterogeneous agricultural landscapes has not been a subject of extensive research. We used occupancy as a measure of jaguar habitat use in Colombia’s middle Magdalena River valley which, as part of the intercontinental Tumbes-Chocó-Magdalena biodiversity hotspot, is exceedingly fragmented by expanding cattle pastures and oil palm plantations. We used single-season occupancy models to analyze 9 months of data (2015–2016) from 70 camera trap sites. Given the middle Magdalena’s status as a “jaguar corridor” and our possible violation of the occupancy models’ demographic closure assumption, we interpreted our results as “probability of habitat use (*Ψ)*” by jaguars. We measured the associations between jaguar presence and coverage of forest, oil palm, and wetlands in radii buffers of 1, 3, and 5 km around each camera trap. Our camera traps recorded 77 jaguar detections at 25 of the camera trap sites (36%) during 15,305 trap nights. The probability of detecting jaguars, given their presence at a site, was 0.28 (0.03 SE). In the top-ranked model, jaguar habitat use was positively influenced by wetland coverage (*β* = 7.16, 3.20 SE) and negatively influenced by cattle pastures (*β* = -1.40, 0.63 SE), both in the 3 km buffers. We conclude that wetlands may serve as keystone habitats for jaguars in landscapes fragmented by cattle ranches and oil palm plantations. Greater focus on wetland preservation could facilitate jaguar persistence in one of the most important yet vulnerable areas of their distribution.

## Introduction

South American rainforests experienced the highest rates of deforestation globally from 2000–2012 [1]. Large carnivores in the Neotropics are especially susceptible to the effects of forest loss and fragmentation due to their occurrence at low densities [2], propensity for conflict with humans [3–4], and dependence on landscape connectivity [5–7]. Yet, empirical data on large carnivore response to habitat loss and fragmentation in the Neotropics is scarce and, in the case of jaguars (*Panthera onca*), most studies have not been conducted in study areas of sufficient size to make robust inferences on the species’ habitat requirements and population parameters [8].

Jaguars are the largest felid in the Americas and the largest terrestrial carnivore in the Neotropics. They favor tropical lowland habitats with sufficient natural cover and access to water and prey [9–11] but the species also inhabits numerous biomes ranging from tropical moist and tropical dry forests to coastal mangroves and herbaceous lowland grasslands [11–12]. Jaguar home ranges vary considerably in relation to prey abundance, habitat quality, and rates of human disturbance [13].

Prey availability is a key determinant of the distribution and abundance of large carnivores [14–15], including jaguars [16–17]. Among terrestrial mammalian species, jaguars tend to prefer capybara (*Hydrochoerus* spp.) and giant anteater (*Myrmecophaga tridactyla*) [18]. White-nosed coati (*Nasua narica*), nine-banded armadillo (*Dasypus novemcinctus*), white-lipped peccary (*Tayassu pecari*), and collared peccary (*Pecari tajacu*) are also frequently recorded in jaguar diet in lowland biomes of the Neotropics [19–22]. In seasonally flooded ecosystems, however, arboreal mammals (i.e. brown-throated sloths *Bradypus variegatus* and red howler monkeys *Alouatta juara*), can be principal prey [17]. In other wetland habitats, species in the reptilian orders of Crocodilia and Testudines dominate both available and consumed biomass [23–26].

Due primarily to habitat loss and poaching, jaguars have been extirpated from approximately 54% of their range, which now spans 18 countries from Mexico to Argentina [6]. The identification of corridors has been a focal strategy in efforts to facilitate connectivity and maintain genetic diversity among jaguar populations (jaguar conservation units, JCUs) [6–7, 9–11]. JCUs are defined as either: (1) areas with a stable prey base and adequate habitat capable of maintaining at least 50 adult jaguars or (2) areas with less than 50 jaguars but with adequate habitat and a stable, diverse prey base that could potentially support an increased jaguar population [11].

Jaguars are considered a vulnerable species in Colombia [27], a critical country within the species’ distribution because it represents part of an intercontinental connection between Mesoamerican and South American JCUs. Embedded in the northeastern portion of the 274,597 km^2^ Tumbes-Chocó-Magdalena hotspot [28], the middle Magdalena River Valley (hereafter, middle Magdalena) is one of the most degraded and least protected biogeographic regions in Colombia [29–30]. It has also long been recognized as a key linkage between jaguar populations east and west of the Andes Mountains [31].

Spanning ~1,291 km^2^, oil palm plantations are increasingly transforming jaguar habitat in the middle Magdalena (S1 Fig; see also [32]). Planted in mono-cropped rows, frequently in industrial-scale plantations that are characterized by high rates of human disturbance and habitat generalists, oil palm is one of the most incompatible land use types for the conservation of tropical biodiversity [33–35]. Colombia is the leading producer of palm oil in Latin America [36] and the middle Magdalena is one of the main areas of production. Information on jaguars in oil palm plantations is limited to studies that did not record jaguars or recorded too few detections to draw conclusions about the species’ response to these vast monocultures [34, 37–38].

Our study evaluated jaguar habitat use in a heterogeneous landscape of the middle Magdalena in an attempt to elucidate the habitat requirements associated with the species’ presence at three spatial scales: 1, 3, and 5 km radii buffers surrounding each camera trap. Since the middle Magdalena is considered a jaguar corridor [39], not a JCU, we sought a better understanding of the probability of our study area being used by jaguars, rather than generating an estimate of their population size. Therefore, we used occupancy as a measure of jaguar habitat use, predicting that jaguar habitat use would increase as the proportion of forest cover and wetlands increased and oil palm and pasture decreased in each buffer size around the camera traps. We further predicted a positive correlation between jaguar detection probability and detections of their major mammalian prey species, defined here as species comprising >0.10 of consumed biomass in jaguar diet in tropical lowland forest and floodplain habitats. These predictions were made on the basis of better access to cover and greater prey availability for jaguars in natural habitats [7, 9, 10, 20–21, 34, 40].

## Materials and methods

### Study area

Located 400 km east of the intercontinental Colombia/Panama border, our study area spanned across ten municipalities within three provinces–Antioquia, Bolívar, and Santander–from 6°36′ to 7°52′ N, -74°22′ to -73°29′ W. The altitudinal range of sampled sites was 40–202 m asl. Mean annual temperature was 27°C and precipitation was 2,500–2,800 mm, and most rainfall occurred in a bimodal pattern from April–May and September–November. There was a distinct dry season from December–February when precipitation averaged less than 130 mm/month. January was the driest month and October was the wettest.

**Fig 1:**
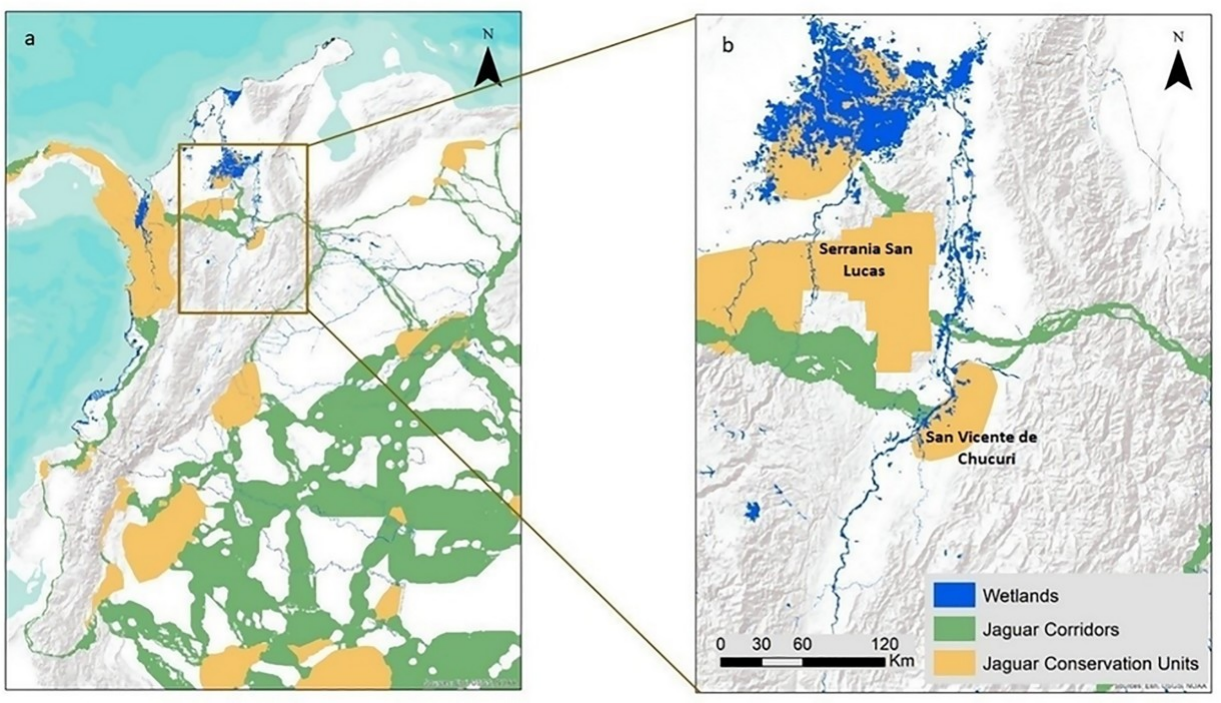
Jaguar corridors and jaguar conservation units in Colombia. Displayed in relation to wetlands and the middle Magdalena River valley (a) and the Serranía San Lucas (b).

The wetlands in our study area consisted of seasonally flooded swamps, permanently flooded marshes, and open-water lakes and rivers [41]. One large forest block existed along the western border of our study area: 27,314 km^2^ of forests encompassed by the Serranía San Lucas (Fig 1). A 3,770 km^2^ portion of the Serranía San Lucas was under evaluation for a new national park in 2014 but extensive mining and occupation by guerilla groups has complicated the declaration process and the park has not yet been formally established.

### Data collection

We used data from camera trap surveys to estimate detection probabilities (*p*) and jaguar occupancy as a measure of their habitat use (*ψ*) from August 2015–April 2016. To minimize the possibility of recording false absences [42] (i.e. where no jaguars are detected despite their home range overlapping a camera trap site), we treated consecutive trap days as repeat surveys at each camera trap site. False absences are major sources of bias in occupancy surveys [43] but they can be differentiated from true absences (i.e. where no jaguars are detected because the camera trap is not placed within a jaguar’s home range) by conducting multiple surveys [42, 44].

**Fig 2:**
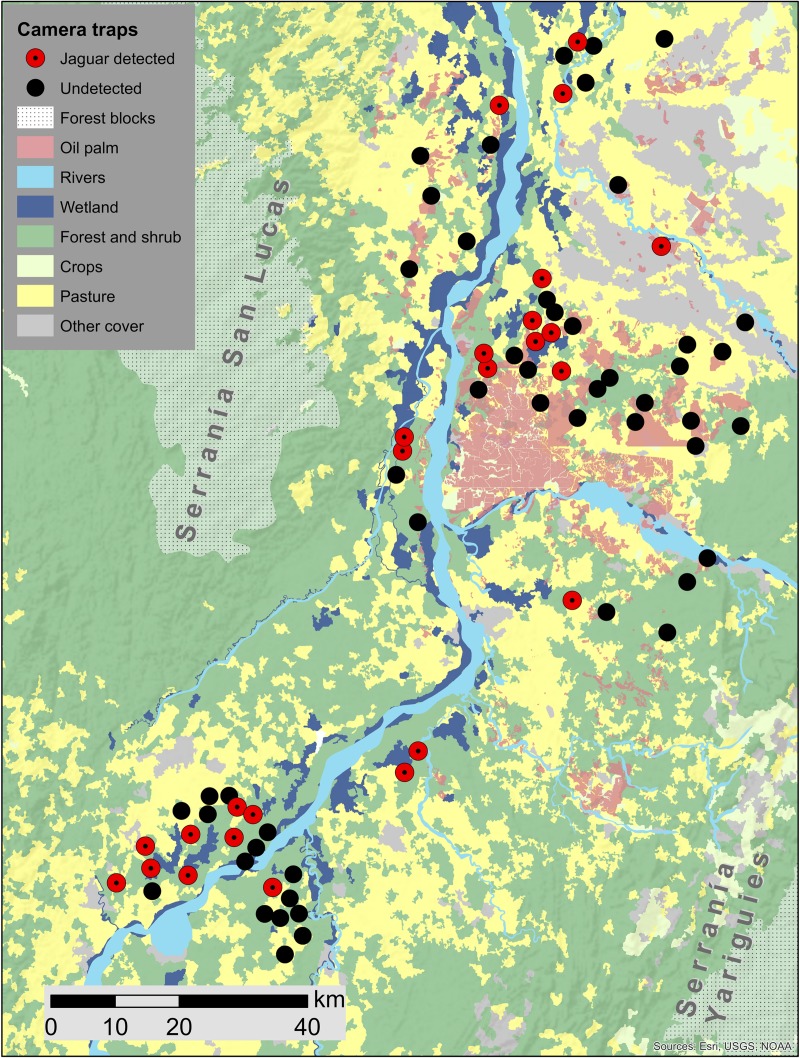
Study area. Land cover and camera trap locations, including sites of jaguar detections in the middle Magdalena River valley, Colombia.

Most of our camera traps were placed on private lands where we required permission for access. A combination of security issues, seasonal flooding, and lack of permission from several large-scale oil palm plantations inhibited our ability to sample some sites southeast of the Serranía San Lucas and northwest of the Serranía de los Yariguíes (Fig 2). The average size of the sampled oil palm plantations was 4.27 km^2^ (range 1.1–9.8 km^2^). The average distance between camera traps and the nearest wetland was 3.54 km (range 0.0–15.5 km). Pasture was the dominant land cover compared to forest, oil palm, and wetland (Table 1).

**Table 1:** Camera trap placement by habitat type. Includes jaguar detections and mean values of habitat covariates at camera trap sites (n = 70) in the middle Magdalena River valley, Colombia.

			Mean (SD) proportion of habitat in radii buffers around camera trapss		
Habitat	Number of camera traps	Number of camera traps with jaguar detections	1 km buffer	3 km buffer	5 km buffer
Forest	20	8	0.23 (0.21)	0.17 (0.13)	0.16 (0.10)
Wetlands	14	11	0.09 (0.24)	0.11 (0.21)	0.12 (0.18)
Pasture	20	4	0.45 (0.40)	0.46 (0.29)	0.45 (0.25)
Oil palm	16	2	0.09 (0.18)	0.10 (0.16)	0.10 (0.15)

Using ArcGIS 9.2 (ESRI, Inc.), we calculated proportions of forest cover, oil palm, pasture, and wetland in radii buffers of 1 km, 3km, and 5 km (3.14, 28.27, and 78.54 km^2^) around each camera trap site (S2 Fig; Table 2). Given the lack of GPS telemetry-based home range estimates from Colombia and the Tumbes-Chocó-Magdalena hotspot [45], we included the multiple buffer sizes to assess jaguar habitat use at varying scales. Species interact with the environment at different scales [46–48] and jaguars exhibit distinct responses to landscape variables at the home range versus foraging scale [49].

**Table 2:** Definitions of covariates (size of radii buffers in km). Summary of covariates used to evaluate jaguar habitat use in the middle Magdalena River valley, Colombia.

Abbreviations	Covariates
for (1, 3, 5)	Forest
wet (1, 3, 5)	Wetland
palm (1, 3, 5)	Oil palm
past (1, 3, 5)	Pasture
prey*	Detections of principal mammalian prey species

*Detection covariate only

We also included detections of principal mammalian prey species as a sampling covariate. We specifically defined these prey species as armadillo, collared peccary, paca (*Cuniculus paca*), lesser capybara (*Hydrochoerus isthmius*), and giant anteater. Collectively, these species dominate the relative occurrence and mammalian biomass of jaguar diet in the lowland Neotropics [19, 20–22]. Our index of prey detections was the number of days on which a principal mammalian prey species was photographed, divided by the total number of trap nights at that site [*sensu* [50].

Prior to running the analyses, we standardized the data using z scores ($x‐\bar{x}/\sigma$). Standardized z-scores can improve model convergence and facilitate the interpretation of the covariate coefficients [51]. Each covariate was selected *a priori* based on our knowledge of jaguar ecology.

### Camera trap surveys

We strategically placed remotely-triggered, passive infrared camera traps (Bushnell Trophy Cam®, Overland Park, KS, and Reconyx® HC500, Holmen, WI) and remotely-triggered, flash camera traps (Cuddeback® Attack, Green Bay, WI and Pantheracam® V4., New York, NY) in paired stations 30–40 cm above the ground. To offset slower trigger speeds of the Bushnell Trophy Cams (~0.6 seconds), we only paired these particular units with the other, faster-triggering, cameras (~0.18–0.30 sec) [52–53]. Average spacing between camera traps was 5.5 km (range 2.9–31.2 km) and camera traps were operational 24 h/day. The minimum convex polygon of our camera trap array covered an area of 7,337 km^2^.

To ensure proper functioning of the camera traps, we revisited our stations every 30–45 days. Camera trap placement was constrained by security issues and lack of permission from the management of several oil palm plantations. Our camera trap survey design was further influenced by seasonal flooding in some inundated areas flanking the Magdalena River. We did not use scents or baits to attract animals. We placed all camera traps off-road because our reconnaissance surveys most commonly recorded jaguar sign on trails and footpaths, which are known travel routes for these cats [54–55]. Although jaguars also frequently use dirt roads for travel [56–57], oil palm workers constantly used dirt roads where risk of theft of our camera traps was a major concern.

### Data analysis

Treating each camera trap site as an individual sampling unit, we analyzed the data in an occupancy framework to estimate the probability of occurrence by incorporating an additional parameter of detection probability [58]. By accounting for detection probability, occupancy models remove the bias resulting from sites where a species was present, but not detected. We defined detection probability as the probability that jaguars were detected in a survey period, given the site was used by jaguars [*sensu* 59]. Single-season models have three key assumptions: (1) The system is demographically closed to changes in occupancy of sites during the sampling period (2) Species are not falsely detected and (3) Detection at a sampling unit (camera trap site) is independent of detection at other sampling units [59].

We constructed detection histories of jaguars for each camera trap site corresponding to a camera trap operational in a 4-week period. This resulted in 9 sampling occasions, which we imported into the program PRESENCE version 2.12.29 [51] to estimate jaguar occupancy (S1 Table). We first modeled detection probability keeping *Ψ* constant and then, under a maximum likelihood framework, applied the top-ranked detection model to the site occupancy models. Covariates were considered to have a significant influence on jaguar occupancy if their 90% CI did not overlap 0. We created individual (univariate) models for each covariate and used AIC to compare them with a null model that did not include covariates. If inclusion of a given covariate improved upon the null model by >2 ΔAIC and the parameter estimate for that covariate did not include zero within a 90% CI, we considered the covariate informative and retained it for the next step of creating multivariate, additive models.

Two occupancy states were possible for each camera trap: occupied (corresponding probability is *Ψ*) and unoccupied (1–*Ψ*). Covariates were incorporated into the occupancy and detection components using the logit-link function, and estimated effect sizes can be interpreted in a similar manner to a logistic regression analysis.

We used Akaike’s information criterion (AIC) corrected for small sample sizes (AICc, n = 70 camera traps) and weighted the support of each model using AICc weights, with lower values indicating greater parsimony [60]. Jaguar detection probabilities were computed as a function of predictor variables using a logit link function. We performed a goodness-of-fit test for single season models to further assess the fit of the selected models [61].

Our data were unable to meet the assumption of population closure as camera traps operated continuously over a 9-month period, during which time the occupancy status of our study area could have varied (i.e. cubs becoming sub-adults and dispersing in or out of the middle Magdalena). Also, given the status of the middle Magdalena as a jaguar corridor [39] not a JCU, we were most interested in the probability of our study area being used by jaguars, rather than true occupancy. Thus, our results should be interpreted as jaguar “probability of habitat use (*Ψ)*” [48, 62].

## Results

Across the 70 sites, the total sampling effort was 15,305 trap nights. We photographed (detected) 12 unique adult jaguars 77 times (6 males, 4 females, and 2 individuals of unknown sex). Jaguars were detected at 25 (36%) of the 70 camera trap stations. We never photographed jaguars at sites without wetlands inside 5 km buffers (n = 16). Three unique females with cubs were photographed at 3 separate camera trap sites during September 2015 and March and April 2016. At sites where we photographed cubs (n = 3), the mean (SD) proportion of habitat in the 1 km radii buffer (3.14 km^2^) around the camera trap stations was 0.79 (0.32) forest, 0.12 (0.21) pasture, and 0.09 (0.12) wetland. Oil palm plantations were absent from the 1 km radii at sites of cub detections.

**Fig 3:**
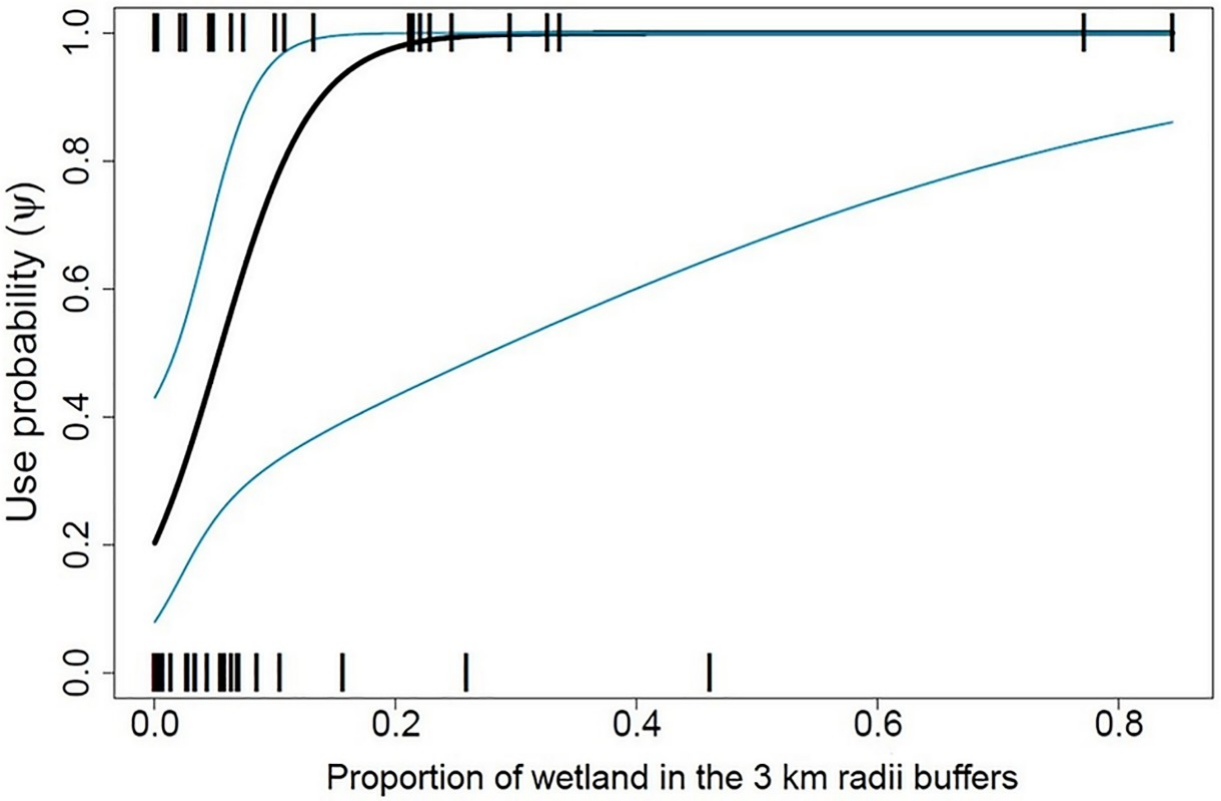
Jaguar habitat use (± 90% CI) as a function of wetland coverage. Displayed at camera trap sites surrounded by 3 km buffers in the middle Magdalena River valley, Colombia.

Jaguar habitat use was most strongly associated with the proportion of wetlands, especially in 3 km buffers (Σw = 0.85) (Tables 3 and 4, Fig 3). The most parsimonious best-fit model for jaguar habitat use–psi(wet3,past3)p(wet3,palm3) (Table 3)–was consistent with our *a priori* expectations of positive associations of jaguar habitat use in buffers with greater spatial extent of wetlands and negative associations with pasture. Jaguar habitat use was also positively associated with the proportion of forest cover, but that association was not significant at any scale.

**Table 3:** Top single-season covariate* models. Ranking of the 14 models evaluated for habitat use of jaguars in the middle Magdalena River valley, Colombia.

Model	AICc	deltaAICc	AIC wgt	Model Likelihood	No. of parameters	Log. likelihood	Cumwt
1.psi(wet3,past3), p(wet3,palm3)	248.05	0.00	0.42	1.00	6	236.05	0.42
2.psi(wet3,palm3,past3),p(wet3,palm3)	248.25	0.20	0.38	0.90	7	234.25	0.80
3.psi(wet5),p(wet5,palm5)	250.36	2.31	0.13	0.32	5	240.36	0.93
4.psi(wet3,palm3),p(wet3,palm3)	253.26	5.21	0.03	0.07	6	241.26	0.96
5.psi(wet3),p(wet3,palm3)	253.73	5.68	0.02	0.06	5	243.73	0.98
6.psi(palm3,past3),p(wet3,palm3)	258.87	10.82	0.01	0.00	6	246.87	0.98
7.psi(past3),p(wet3,palm3)	261.90	13.85	0.00	0.00	5	251.90	0.98
8.psi(wet1),p(wet1)	264.05	16.00	0.00	0.00	4	256.05	0.99
9.psi(palm3),p(wet3,palm3)	264.14	16.09	0.00	0.00	5	254.14	0.99
10.psi(for5),p(wet5,palm5)	264.77	16.72	0.00	0.00	5	254.77	0.99
11.psi(.),p(wet3,palm3)	267.26	19.21	0.00	0.00	4	259.26	1.00
12.psi(.),p(wet5,palm5)	268.44	20.39	0.00	0.00	4	260.44	1.00
13.psi(.),p(wet1)	273.21	25.16	0.00	0.00	3	267.21	1.00
14.psi(.),p(.)	274.57	26.52	0.00	0.00	2	270.57	1.00

*Site and sampling covariates: wet1 = percentage of wetland coverage in 1 km buffers around each camera trap, wet3 = percentage of wetland coverage in 3 km buffers around each camera trap, palm3 = percentages of oil palm coverage in 3 km buffers around each camera trap, for5 = percentage of forest cover in 5 km buffers around each camera trap.

**Table 4:** Results of the top models. Parameter estimates and 90% credible intervals (CI) influencing jaguar habitat use in the middle Magdalena River valley, Colombia. Covariates were considered to have a significant influence on jaguar habitat use when their 90% CI did not overlap zero (marked in bold).

Models	β int (90% CI)	βwet5 (90% CI)	βwet3 (90% CI)	βpalm3 (90% CI)	βpast3 (90% CI)
Model1	2.02 (-0.23,4.27)	-	**7.16 (1.89,12.43)**	**-**	**-1.40 (-0.37,-2.43)**
Model2	0.70 (-0.91,2.05)	-	**4.94** **(1.33,8.55)**	**-1.32** **(-0.09,-2.55)**	**-1.36** **(-0.33,-2.39)**
Model3	7.14 (-1.99, 16.27)	15.38 (-0.97, 31.73)	-	-	-
Model4	-0.13 (-1.23, 1.07)	-	**2.76 (0.47, 5.05)**	**-1.21** **(-2.27,-0.15)**	-

**Fig 4:**
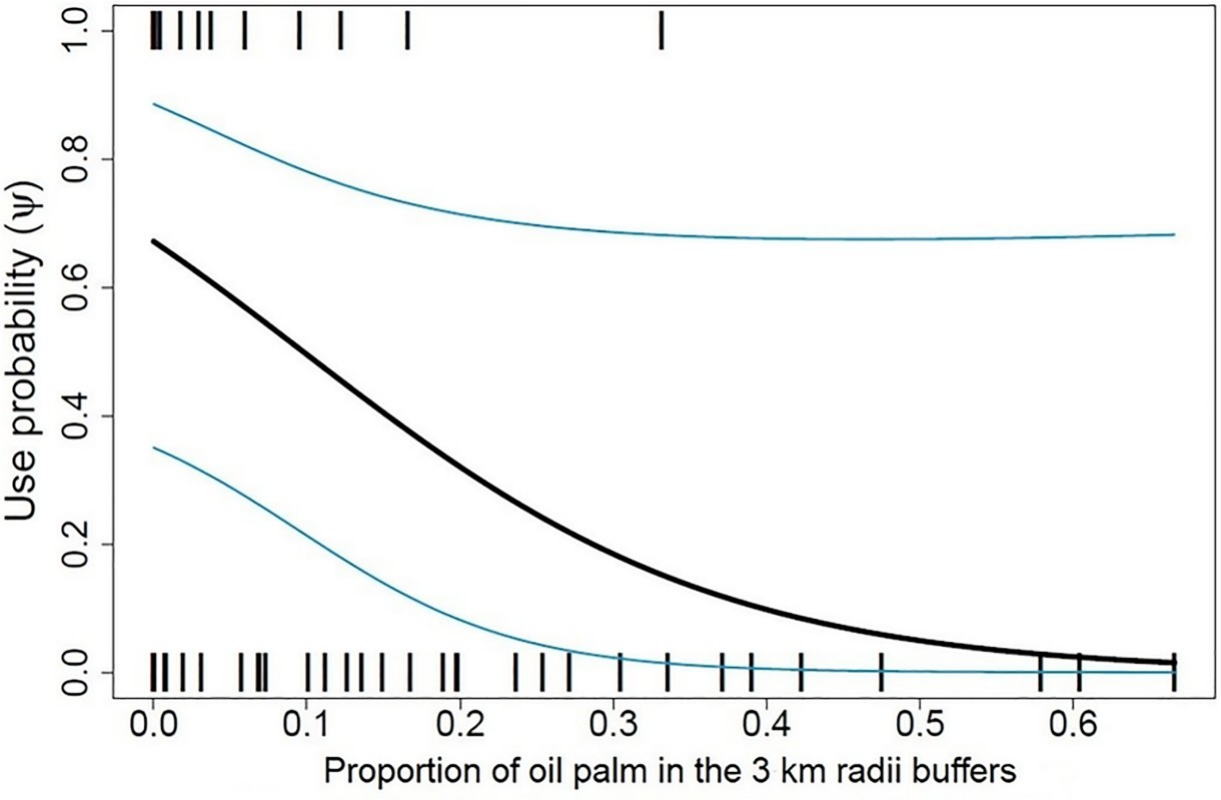
Jaguar habitat use (± 90% CI) as a function of oil palm coverage. Displayed at camera trap sites surrounded by 3 km buffers in the middle Magdalena River valley, Colombia.

Jaguar habitat use was negatively correlated with the proportion of pasture (*β* = -1.40, 0.63 SE) and oil palm (*β* = -1.21, 0.65 SE). Once extent of plantation coverage reached ~50% of the area inside 3 km buffers around camera traps, jaguar habitat use declined significantly (Fig 4). Our goodness-of-fit test for the most parameterized multivariate model revealed no evidence of overdispersion (lack of independence), suggesting the models provided an adequate description of the data (Model 2: χ^2^ = 255.10, P = 0.97, ĉ = 0.40).

The probability of detecting jaguars, given their presence at a site, was 28% (0.03 SE). There was very strong evidence that detection probabilities were positively influenced by wetland coverage in 3 km buffers (*β* = 0.36, 0.14 SE) and negatively influenced by oil palm plantations in 3 km buffers (*β* = -0.75, 0.33 SE). Contrary to expectation, there was a negative association between detections of jaguars and their principal mammalian prey (*β* = -0.71, 0.40 SE).

Peccary and paca were the most frequently detected prey species and were also recorded at the greatest number of camera traps (Table 5). Giant anteaters and paca were most frequently detected in wetland habitats. Armadillo, capybara, and giant anteater were not detected in oil palm plantations. However, insufficient giant anteater and capybara detections prevented detailed analyses for these two species.

**Table 5:** Summary of prey detections. Prey detections by habitat type and mean values of habitat covariates in 1 km radii buffers at camera trap sites (n = 70) in the middle Magdalena River valley, Colombia.

				Mean (SD) proportion of habitat in 1 km radii buffers around camera traps			
Species	Number of camera traps with detections	Naïve occupancy	Total detections	Forest	Wetland	Oil palm	Pasture
Collared peccary	21	0.30	76	0.33 (0.22)	0.03 (0.07)	0.08 (0.25)	0.25 (0.23)
Paca	19	0.27	76	0.29 (0.24)	0.08 (0.23)	0.02 (0.06)	0.29 (0.27)
Armadillo	15	0.21	44	0.30 (0.23)	0.01 (0.05)	0.00 (0.00)	0.37 (0.31)
Capybara	4	0.06	16	0.10 (0.11)	0.00 (0.00)	0.00 (0.00)	0.56 (0.37)
Giant anteater	4	0.06	10	0.17 (0.13)	0.19 (0.26)	0.00 (0.00)	0.62 (0.43)

## Discussion

Despite their well-documented affinity for habitats near permanent surface water [9, 19, 40, 57, 63–65] this is one of the first studies to quantify associations between wetlands and jaguar habitat use. Wetlands in the Brazilian Pantanal and in the Venezuelan llanos are known strongholds for jaguars [9, 20, 63, 66] but previous studies in these areas were not specifically designed to identify associations between habitat features and jaguar presence in landscapes heavily modified by agriculture.

Notably, jaguar habitat use in the middle Magdalena was most strongly associated with the intermediate spatial scale (3-km buffer). This scale (28.27 km^2^) most closely corresponds to the size of a female jaguar home range size in seasonally flooded habitats [13]. Considering the lack of home range estimates from both Colombia and the Tumbes-Chocó-Magdalena hotspot [45], the 3-km scale could be the most accurate representation of the utilization of habitats present within the home range of a female jaguar in the middle Magdalena. Selection for preferred habitats (i.e. forests for hiding and raising cubs and wetlands for foraging) will influence how jaguars move in relation to these landscape components and the scale at which they affect habitat use [49]. For example, compared to the mean proportion of habitats across our study area, the proportions at sites of cub detections (n = 3) included similar wetland coverage but significantly greater forest cover and less disturbed habitats (oil palm and pasture).

Nonetheless, the clear lack of support for forest cover in our models suggests that jaguars may be less dependent on forests in areas dominated by wetlands or they may preferentially select wetlands in areas lacking forest cover. Across eight study areas in Brazil and Argentina, jaguars showed increasingly strong selection for forests in landscapes with >58% forest cover but they showed less avoidance of non-forest areas at sites with greater proportions of open and deforested areas [49].

Excluding the Serranía San Lucas JCU, which is the largest contiguous block of forest in our study area (27,314 km^2^), 93% of natural habitats in the middle Magdalena had been converted to agriculture by 2006 [29]. Wetlands could provide increasingly important habitat for jaguars in degraded landscapes of the middle Magdalena, because the Serranía San Lucas experienced the fourth-greatest extent of habitat loss among JCUs range-wide, losing 1,590 km^2^ (5.82%) of its forest cover from 2000–2012 [39].

Given the oil palm industry’s demanding water footprint of 5,000 m^3^ ton^–1^ [67], wetlands are now at greater risk to draining in the middle Magdalena, which was recently identified as one of 14 hotspots of wetland loss in Colombia [68]. Beyond the middle Magdalena, one of the primary zones targeted for oil palm expansion is the tropical savannah of the Orinoco region [69–70], an ecoregion that contains both 55% of Colombia’s wetlands [71] and the Orinoco-Amazon JCU [11]. Wetlands in this region, as elsewhere in Colombia, lack protection.

The Ramsar Convention, which requires a national policy for the management and protection of wetlands and their biodiversity, has resulted in the establishment of only five Ramsar sites in Colombia. A Ramsar site is defined as ‘a wetland of international importance that contains representative, rare or unique wetland types important for maintaining biodiversity, supporting threatened species or communities and providing refuge during adverse conditions in a particular biogeographic region’ [72]. The Estrella Fluvial Inírida is the only Ramsar site in Colombia’s Orinoco ecoregion, where jaguars are present [37]. Ramsar sites are entirely absent from the middle Magdalena.

Around the world, Ramsar sites provide important habitat for large carnivores besides jaguars. These protected wetlands overlap several key tiger (*Panthera tigris*) conservation units such as the Sunderbans in Bangladesh and the Beeshazar region of the Terai landscape in Nepal [73]. In the Terai, tiger occupancy is positively correlated with wetlands, particularly the highly productive alluvial floodplains and riverine forests, both of which harbor high densities of ungulate prey [74–75]. Similarly, the Osceola National Forest-Okefenokee Swamp ecosystem provides important foraging habitat for one of the largest remaining black bear (*Ursus americanus*) populations in the southeastern USA [76].

The lack of a positive correlation between detections of jaguars and their terrestrial mammalian prey raises hypotheses about wetlands as important foraging areas for jaguars. Across our study area, the ratio of detections of jaguars and principal mammalian prey was 1:1 for peccary and paca, 1:0.57 for armadillo, 1:0.21 for capybara, and 1:0.13 for giant anteater. These ratios are suggestive of a depleted mammalian prey base because camera trap surveys–even those targeting jaguars–generally record more detections of prey than jaguars [16, 34, 55].

In wetlands, significant proportions of jaguar diet may be comprised of aquatic and semi-aquatic species [23, 25–26, 77]. In the middle Magdalena, potential reptilian prey include spectacled caiman (*Caiman crocodilus*), American crocodiles (*Crocodylus acutus*), Magdalena River turtles (*Podocnemis lewyana*) and Colombian slider turtles (*Trachemys callirostris*). In flooded forests of the Amazon basin, spectacled caimans were recorded in 41% of jaguar scat samples [78] and reptiles comprised 36% of jaguar diet in the floodplains of the San Jorge and Cauca rivers [24], which are located 150 km northwest of our study area. We observed evidence of jaguar depredation on Colombian sliders in the middle Magdalena where this species is widely distributed [79].

We recommend future studies in wetland areas examine the importance of reptilian prey, which were undetected by our terrestrial, heat-sensitive camera traps. Also, we suggest finer-scale analyses that differentiate jaguar habitat use of specific wetland habitat types (i.e. marshes, swamps, and floodplain forests). Globally, there are significant data gaps for the spatial extent of wetland classes–particularly for lower order streams, ponds, and marshes [80]–but recent advancements have contributed to improved inventories at regional scales [81].

Finally, we stress the importance of long-term monitoring to evaluate trends in jaguar habitat use and occupancy in the middle Magdalena. As the proportion of oil palm and pasture increases relative to forests and wetlands, the habitat is likely to become less suitable for jaguars. For example, recent camera trap surveys did not detect tigers in oil palm landscapes of lowland Peninsular Malaysia [82–84], which is one of their historical strongholds [85–86]. Globally, most palm oil is produced in Malaysia and Indonesia where its destructive impacts on threatened species have been well documented [87–90].

With oil palm cultivation projected to increase in Latin America–including Colombia [69–70]–there is greater urgency to collect data on jaguars in oil palm landscapes to guide the identification and implementation of appropriate land use planning and zoning measures. Our results suggest that jaguars may tolerate oil palm smallholdings but avoid large-scale plantations because habitat use declined significantly once extent of plantation coverage reached ~50% of the area inside 3 km buffers around camera traps. Jaguar avoidance of oil palm plantations is likely a response to the depauperate prey bases and greater rates of human disturbance in these monocultures [33–35, 87].

## Conclusion

Evaluation of occupancy and habitat use at large spatial scales is necessary for identifying the ecological needs of wide-ranging species [8, 91]. To our knowledge this study is one of the largest, in terms of spatial coverage and sampling effort, to survey jaguars with camera traps using an occupancy approach. On the basis of our results, we propose that wetlands receive greater consideration as keystone habitats for jaguars. Keystone habitats have disproportionately large effects relative to their availability and contain resources (i.e. reptilian prey for jaguars) not provided by other available habitats [92]. Wetlands comprise ~30% of the Amazon basin [93] where 16 Ramsar sites span some 342,084 km^2^, 4.9% of the entire basin. Beyond the Amazon basin–which is the jaguar’s range-wide stronghold [10–11, 39]–favorable policies for wetland conservation are grossly lacking [93–95]. For example, there are only 11 Ramsar sites spanning some 1,330 km^2^ in the Tumbes-Chocó-Magdalena hotspot [S2 Table]. Greater protection of wetlands and riparian buffers–which is already required under Colombian law (Resolution No. 200.41.11–1130, 2011) [96]–could facilitate jaguar persistence in fragmented landscapes and areas undergoing oil palm and pasture development.

## Supporting information

S1 Fig Overlap between oil palm plantations and modeled jaguar corridors in Colombia. (TIF)

S2 Fig Example showing the radii buffers of 1 km, 3km, and 5 km (3.14, 28.27, and 78.54 km^2^) around camera trap site. (TIF)

S1 Table Jaguar detection matrix uploaded to PRESENCE version 12.17. (XLSX)

S2 Table Ramsar sites in the Tumbes-Chocó-Magdalena biodiversity hotspot. (XLSX)

## References

[1] Hansen MC, Potapov PV, Moore R, Hancher M, Turubanova SA, Tyukavina A, et al High resolution global maps of 21^st^ century forest cover change. Science. 2013; 342: 850–853. 10.1126/science.1244693 24233722

[2] Ripple WJ, Estes JA, Beschta RL, Wilmers CC, Ritchie EG, Hebblewhite M, et al Status and ecological effects of the world’s largest carnivores. Science. 2014; 343: 151–163. 10.1126/science.1241484 24408439

[3] Treves A, Karanth KU. Human-carnivore conflict and perspectives on carnivore management worldwide. Conserv Biol. 2003; 17: 1491–1499.

[4] Goldstein I, Paisley S, Wallace RB, Jorgenson JP, Cuesta F, Castellanos A. Andean bear-livestock conflicts: A review. Ursus. 2006; 17: 8–15.

[5] Kattan G, Lucía-Hernández O, Goldstein I, Rojas V, Murillo O, Gómez C, et al Range fragmentation of the spectacled bear in the northern Andes. Oryx. 2004; 38: 155–163.

[6] Rabinowitz A, Zeller K. A range-wide model of landscape connectivity and conservation for the jaguar. Biol Conserv. 2010; 143: 939–945.

[7] de la Torre JA, Núñez JM, Medellín RA. Habitat availability and connectivity for jaguars in the Southern Mayan Forest: Conservation priorities for a fragmented landscape. Biol Conserv. 2017; 206: 270–282.

[8] Tobler MW, Powell, GVN. Estimating jaguar densities with camera traps: Problems with current designs and recommendations for future studies. Biol Conserv. 2013; 159: 109–118.

[9] Quigley HB, Crawshaw Jr. PG. A conservation plan for the jaguar in the Pantanal region of Brazil. Biol Conserv. 1992; 61: 149–157.

[10] Silveira L, Sollmann R., Jácomo ATA, Diniz-Filho JAF, Torres NM. The potential for large-scale wildlife corridors between protected areas in Brazil using the jaguar as a model species. Landsc Ecol. 2014; 29: 1213–1223.

[11] Sanderson EW, Redford KH, Chetkiewicz CB, Medellin RA, Rabinowitz A, Robinson JG, et al Planning to save a species: the jaguar as a model. Conserv Biol. 2002; 16: 1–15. 10.1046/j.1523-1739.2002.00352.x 35701976

[12] Zeller K. Jaguars in the new millennium data set update: the state of the jaguar in 2006. New York: Wildlife Conservation Society; 2007.

[13] Morato RG, Stabach JA, Fleming CH, Calabrese JM, de Paula RC, Ferraz KMPM, et al Space use and movement of a Neotropical top predator: the endangered jaguar. PLoS ONE. 2016; 11: e0168176 10.1371/journal.pone.0168176 28030568 PMC5193337

[14] Karanth KU, Nichols JD, Kumar NS, Link WA, Hines JE. Tigers and their prey: predicting carnivore densities from prey abundance. Proc Natl Acad Sci U.S.A. 2004; 101: 4854–4858. 10.1073/pnas.0306210101 15041746 PMC387338

[15] Duangchatrasiri S, Jornburom P, Jinamoy S, Pattanvibool A, Hines JE, Arnold TW, et al Impact of prey occupancy and other ecological and anthropogenic factors on tiger distribution in Thailand’s western forest complex. Ecol Evol. 2019; 9: 2449–2458. 10.1002/ece3.4845 30891192 PMC6405490

[16] Santos F, Carbone C, Wearn OR, Rowcliffe JM, Espinosa S, Lima MGM, et al Prey availability and temporal partitioning modulate felid coexistence in Neotropical forests. PLoS ONE. 2019; 14: e0213671 10.1371/journal.pone.0213671 30861045 PMC6413900

[17] Rabelo RM, Aragon S, Bicca-Marques JC. Prey abundance drives habitat occupancy by jaguars in Amazonian floodplain river islands. Acta Oecol. 2019; 97: 28–33.

[18] Hayward MW, Kamler JF, Montgomery RA, Newlove A, Rostro-Garcia S, Sales LP, et al Prey preferences of the jaguar reflect the post-Pleistocene demise of large prey. Front Ecol Evol. 2016; 3: 148.

[19] Emmons LH. Comparative feeding ecology of felids in a Neotropical forest. Behav Ecol Sociobiol. 1987; 20: 271–283.

[20] Scognamillo D, Maxit IE, Sunquist M, Polisar J. Coexistence of jaguar and puma in a mosaic landscape in the Venezuelan llanos. J Zool. 2003; 259: 269–279.

[21] Foster RJ, Harmsen BJ, Valdes B, Pomilla C, Doncaster CP. Food habits of sympatric jaguars and pumas across a gradient of human disturbance. Biotropica. 2010; 280: 309–318.

[22] Cavalcanti SMC, Gese EM. Kill rates and predation patterns of jaguars in the southern Pantanal, Brazil. J Mamm. 2010; 91: 722–736.

[23] Emmons LH. Jaguar predation on chelonians. J Herpetol. 1989; 23: 311–314.

[24] Zuloaga JG. Densidad de población, hábitos alimenticios y anotaciones sobre habitat natural del jaguar en la depression inundable del bajo San Jorge, Colombia Título de Biólogo, Universidad Nacional de Colombia, Bogotá 1995.

[25] Da Silveira F, Ramalho EE, Thorbjarnarson J, Magnusson W. Depredation by jaguars on caimans and importance of reptiles in the diet of jaguar. J Herpetol. 2010; 44: 418–424.

[26] Azevedo FCC, Verdade LM. Predator-prey interactions: jaguar predation on caiman in a floodplain forest. J Zool. 2012; 286: 200–207.

[27] Rodríguez-Mahecha JV, Jorgenson JP, Duran-Ramirez C, Bedoya-Gaitán M. Jaguar Panthera onca In: Rodriguez JV, Alberico M, Trujillo F, Jorgenson J, editors. Libro Rojo de los Mamíferos de Colombia. Bogotá: Conservación Internacional; 2006 pp. 260–265.

[28] Mittermeier RA, Turner WR, Larsen FW, Brooks TM, Gascon C. Global biodiversity conservation: the critical role of hotspots In: Zachos FE, Habel JC, editors. Biodiversity hotspots: distribution and protection of conservation priority areas. Heidelberg: Springer; 2011 p. 3–22.

[29] Etter A, McAlpine C, Wilson K, Phinn S, Possingham H. Regional patterns of agricultural land use and deforestation in Colombia. Agric Ecosyst Environ. 2006; 114: 369–386.

[30] Forero-Medina G, Joppa L. Representation of global and national conservation priorities by Colombia’s protected area network. PLoS ONE. 2010: e13210 10.1371/journal.pone.0013210 20967270 PMC2953503

[31] Melquist WE. Status survey of otters and spotted cats in Latin America IUCN Report: 45–253. Idaho Cooperative Wildlife Research Unit. Moscow: University of Idaho; 1984.

[32] FEDEPALMA. Anuario Estadístico 2014. La agroindustria de la palma de aceite en Colombia y en el mundo: 2009–2013 Bogotá: FEDEPALMA; 2014.

[33] Yue S, Brodie JF, Zipkin EF, Bernard H. Oil palm plantations fail to support mammal diversity. Ecol Appl. 2015; 25: 2285–2292. 26910955 10.1890/14-1928.1

[34] Mendes-Oliveira AC, Peres CA, Maués P, Oliveira GL, Mineiro IGB, de Maria SLS, et al Oil palm monoculture induces drastic erosion of an Amazonian forest mammal fauna. PLoS ONE. 2017; 12: e0187650 10.1371/journal.pone.0187650 29117202 PMC5695600

[35] Meijaard E, Garcia-Ulloa J, Sheil D, Wich SA, Carlson KM, Juffe-Bignoli D, et al Oil palm and biodiversity A situation analysis by the IUCN Oil Palm Task Force. Gland Switzerland: IUCN; 2018.

[36] FAO. FAOSTAT Online Statistical Service. Food and Agricultural Organization of the United Nations. 2016. Available from http://faostat3.fao.org (accessed December 2018)

[37] Borron V, Tzanopoulos J, Gallo J, Barragan J, Jaimes-Rodriguez L, Schaller GB, et al Jaguar densities across human-dominated landscapes in Colombia: the contribution of unprotected areas to long term conservation. PLoS ONE. 2016; 11: e0153973 10.1371/journal.pone.0153973 27144280 PMC4856405

[38] Pardo LE, Campbell MJ, Edwards W, Clements GR, Laurance WF. Terrestrial mammal responses to oil palm dominated landscapes in Colombia. PLoS ONE. 2018; 13: e0197539 10.1371/journal.pone.0197539 29795615 PMC5968401

[39] Olsoy PJ, Zeller KA, Hicke JA, Quigley HB, Rabinowitz AR, Thornton DH. Quantifying the effects of deforestation and fragmentation on a range-wide conservation plan for jaguars. Biol Conserv. 2016; 203: 8–16.

[40] Foster RJ, Harmsen BJ, Doncaster CP. Habitat use by sympatric jaguars and pumas across a gradient of human disturbance in Belize. Biotropica. 2010; 42: 724–731.

[41] IDEAM. Zonificación Hidrográfica de Colombia, Escala 1:2.250.000. Bogota. 2013.

[42] Mackenzie DI, Royle JA. Designing occupancy studies: General advice and allocating survey effort. J Appl Ecol. 2005; 42: 1105–1114.

[43] Moilanan A. Implications of empirical data quality for metapopulation model parameter estimation and application. Oikos. 2002; 96: 516–530.

[44] Linkie M, Chapron G, Martyr DJ, Holden J, Leader-Williams N. Assessing the viability of tiger subpopulations in a fragmented landscape. J Appl Ecol. 2006; 43: 576–586.

[45] Morato RG, Thompson JJ, Paviolo A, de la Torre JA, Lima F, McBride RT, et al Jaguar movement database: a GPS-based movement dataset of an apex predator in the Neotropics. Ecology. 2018; 99: 1691 10.1002/ecy.2379 29961270

[46] Ciarniello LM, Boyce MS, Seip DR, Heard DC. Grizzly bear habitat selection is scale dependent. Ecol Appl. 2007; 17: 1424–1440. 17708219 10.1890/06-1100.1

[47] Pusparini W, Sievert PR, Fuller TK, Randhir TO, Andayani N. Rhinos in the parks: an island-wide survey of the last wild population of the Sumatran rhinoceros. PLoS ONE. 2015; 10: e0139982 10.1371/journal.pone.0139982 26376453 PMC4574046

[48] Nagy-Reis MB, Nichols JD, Chiarello AG, Ribeiro MC, Setz EZF. Landscape use and co-occurrence patterns of Neotropical spotted cats. PLoS ONE. 2016; 12: e0168441. 10.1371/journal.pone.0168441 PMC5215768 28052073

[49] Morato RG, Connette GM, Stabach JA, de Paula RC, Ferraz KMPM, Kantek DLZ, et al Resource selection in an apex predator and variation in response to local landscape characteristics. Biol Conserv. 2018; 228: 233–240.

[50] Alexander JS, Shi K, Tallents L, Riordan P. On the high trail: examining determinants of site use by the endangered snow leopard in Qilianshan, China. Oryx. 2016; 50: 231–238.

[51] Hines JE. 2010. Program PRESENCE (Version 12.17). <http://www.mbr-pwrc.usgs.gov/software/doc/presence/presence.html>.

[52] Rovero F, Zimmermann F, Berzi D, Meek P. “Which camera trap type and how many do I need?” A review of camera features and study designs for a range of wildlife research applications. Hystrix. 2013; 24: 148–156.

[53] Olliff ERR, Cline CW, Bruen DC, Yarmchuk EJ, Pickles RSA, Hunter L. The Pantheracam–a camera trap optimized for monitoring wild felids. Wild Felid Monitor 2014; 7: 21–23.

[54] Harmsen BJ, Foster RJ, Silver S, Ostro L, Doncaster CP. Differential use of trails by forest mammals and the implications for camera trap studies. Biotropica 2010; 42: 126–133.

[55] Tobler MW, Zuñiga Hartley A, Carrillo-Percastegui SE, Powell GVN. 2015. Spatiotemporal hierarchical modelling of species richness and occupancy using camera trap data. J Appl Ecol. 2015; 52: 413–421.

[56] Sollmann R, Furtado MM, Hofer H, Jácomo ATA, Torres NM, Silveira L. Using occupancy models to investigate space partitioning between two sympatric large predators, the jaguar and puma in central Brazil. Mamm Biol. 2012; 77: 41–46.

[57] Figel JJ, Ruíz-Gutiérrez F, Brown DE. Densities and perceptions of jaguars in coastal Nayarit, Mexico. Wildl Soc Bull. 2016; 40: 506–513.

[58] MacKenzie DI, Nichols JD, Lachman GB, Droege S, Royle JA, Langtimm CA. Estimating site occupancy rates when detection probabilities are less than one. Ecology. 2002; 83: 2248–2255.

[59] MacKenzie DI, Nichols JD, Royle JA, Pollock KH, Bailey LL, Hines JE. Occupancy estimation and modeling: inferring patterns and dynamics of species occurrence. New York: Academic Press; 2006.

[60] Burnham KP, Anderson DR. Model selection and multimodel inference: a practical information-theoretic approach. 2nd edn New York: Springer-Verlag; 2002.

[61] MacKenzie DI, Bailey LL. Assessing the fit of site-occupancy models. J Agric Biol Environ Stat. 2004; 9: 300–318.

[62] MacKenzie DI, Nichols JD. Occupancy as a surrogate for abundance estimation. Anim Biodivers Conserv. 2004; 27: 461–467.

[63] Crawshaw Jr PG, Quigley HB. Jaguar spacing, activity and habitat use in a seasonally flooded environment in Brazil. J Zool. 1991; 222: 357–370.

[64] Zeller K, Nijhawan S, Salom-Pérez R, Potosme SH, Hines JE. Integrating occupancy modeling and interview data for corridor identification: a case study for jaguars in Nicaragua. Biol Conserv. 2011; 144: 892–901.

[65] Cullen L Jr, Sana DA, Lima F, de Abreu KC, Uezu A. Selection of habitat by the jaguar in the upper Paraná river, Brazil. Zoologia. 2013; 30: 379–387.

[66] Soisalo MK, Cavalcanti SMC. Estimating the density of a jaguar population in the Brazilian Pantanal using camera-traps and capture-recapture sampling in combination with GPS radio-telemetry. Biol Conserv. 2006; 129: 487–496.

[67] Mekonnen MM, Hoekstra AY. The green, blue and grey water footprint of crops and derived crop products. Hydrol Earth Syst Sci. 2011; 15: 1577–1600.

[68] Patino JE, Estupinan-Suarez LM. Hotspots of wetland area loss in Colombia. Wetlands. 2016; 36: 935–943.

[69] Garcia-Ulloa J, Sloan S, Pacheco P, Ghazoul J, Koh LP. Lowering environmental costs of oil palm expansion in Colombia. Conserv Lett. 2012; 5: 366–375.

[70] Ocampo-Peñuela N, Garcia-Ulloa J, Ghazoul J, Etter A. Quantifying impacts of oil palm expansion on Colombia’s threatened biodiversity. Biol Conserv. 2018; 224: 117–121.

[71] IDEAM, IGAC, IAvH, Invemar, I Sinchi, IIAP. Ecosistemas continentales, costeros y marinos de Colombia Bogotá: Instituto Geográfico Agustín Codazzi (IGAC); 2007.

[72] Ramsar Convention Secretariat. The Ramsar Convention and its mission. Ramsar Conservation Secretariat, Gland, Switzerland. 2014.

[73] Sanderson EJ, Forrest J, Loucks C, Ginsberg J, Dinerstein E, Seidensticker J, et al Setting priorities for the conservation and recovery of wild tigers: 2005–2015. WCS, WWF, Smithsonian, NFWF-STF New York–Washington DC 2006.

[74] Chanchani P, Noon BR, Bailey L, Warrier RA. Conserving tigers in working landscapes. Conserv Biol. 2016; 30: 649–660. 10.1111/cobi.12633 26400445

[75] Lamichhane BR, Leirs H, Persoon GA, Subedi N, Dhakal M, Oli BN, et al Factors associated with co-occurrence of large carnivores in a human-dominated landscape. Biodiversity Conserv 2019; 28: 1473–1491.

[76] Dobey S, Masters DV, Scheick BK, Clark JD, Pelton MR, Sunquist ME. Ecology of Florida black bears in the Okefenokee-Osceola ecosystem. Wildl Monogr. 2005; 158: 1–41.

[77] Schaller GB, Vasconcelos JMC. Jaguar predation on capybara. Zeitschrift für Säugetierkunde. 1978; 43: 296–301.

[78] Ramalho EE. Jaguar population dynamics, feeding ecology, human induced mortality, and conservation in the Varzea floodplain forests of Amazonia. PhD dissertation, University of Florida. 2012.

[79] Bock BC, Páez VP, Daza JM. Trachemys callirostris (Gray 1856)–Colombian slider, jicotea, hicotea, galapago, morrocoy de agua. Chelonian Research Monographs. 2010; 5: 042.1–042.9.

[80] Davidson NC, Finlayson CM. Extent, regional distribution and changes in area of different classes of wetland. Mar Freshwater Res. 2018; 69: 1525–1533.

[81] Gumbricht T, Roman-Cuesta RM, Verchot L, Herold M, Wittmann F, Householder E, et al An expert system model for mapping tropical wetlands and peatlands reveals South America as the largest contributor. Glob Change Biol. 2017; 23: 3581–3599. 10.1111/gcb.13689 28295834

[82] Sasidhran S, Adila N, Hamdan MS, Samantha LD, Aziz N, Kamarudin N, et al Habitat occupancy patterns and activity rate of native mammals in tropical fragmented peat swamp reserves in Peninsular Malaysia. Forest Ecol Manag. 2016; 363: 140–148.

[83] Adila N, Sasidhran S, Kamarudin N, Puan CL, Azhar B, Lindenmayer DB. Effects of peat swamp logging and agricultural expansion on species richness of native mammals in Peninsular Malaysia. Basic Appl Ecol. 2017; 22: 1–10.

[84] Jamhuri J, Samantha LD, Tee SL, Kamarudin N, Ashton-Butt A, Zubaid A, et al Selective logging causes the decline of large-sized mammals including those in unlogged patches surrounded by logged and agricultural areas. Biol Conserv. 2018; 227: 40–47.

[85] Locke A. The tigers of Terengganu. London, UK: Museum Press Ltd; 1954.

[86] Khan MKM. Tiger in Malaysia: prospects for the future In: Tilson RC, Seal US, editors. Tigers of the world. Park Ridge, New Jersey: Noyes Publications; 1987 p. 75–84.

[87] Maddox T, Priatna D., Gemita E, Salampessy A. The conservation of tigers and other wildlife in oil palm plantations. London: Zoological Society of London; 2007.

[88] Sunarto S, Kelly MJ, Parakkasi K, Klenzendorf S, Septayuda E, Kurniawan H. Tigers need cover: multi-scale occupancy study of the big cat in Sumatran forest and plantation landscapes. PLoS ONE. 2012; 7: e30859 10.1371/journal.pone.0030859 22292063 PMC3264627

[89] Alcrenaz M, Oram F, Ambu L, Lackman I, Ahmad E, Elahan H, et al Of Pongo, palms and perceptions: a multidisciplinary assessment of Bornean orang-utans Pongo pygmaeus in an oil palm context. Oryx. 2015; 49: 465–472.

[90] Sulai P, Nurhidayu S, Aziz N, Zakaria M, Barclary H, Azhar B. Effects of water quality in oil palm production landscapes on tropical waterbirds in Peninsular Malaysia. Ecol Res. 2015; 30: 941–949.

[91] Karanth KU, Gopalaswamy AM, Kumar NS, Vaidyanathan S, Nichols JD, MacKenzie DI. Monitoring carnivore populations at the landscape scale: occupancy modelling of tigers from sign surveys. J Appl Ecol. 2011; 48: 1048–1056.

[92] Hitchman SM, Mather ME, Smith JM, Fencl JS. Identifying keystone habitats with a mosaic approach can improve biodiversity conservation in disturbed ecosystems. Glob Change Biol. 2018; 24: 308–321. 10.1111/gcb.13846 28755429

[93] Junk WJ, Piedade MTF, Schöngart J, Cohn-Haft M, Adeney JM, Wittmann F. A classification of major naturally-occurring Amazonian lowland wetlands. Wetlands. 2011; 31: 623–640.

[94] Junk WJ. Current state of knowledge regarding South America wetlands and their future under global climate change. Aquat Sci. 2013; 75: 113–131.

[95] Sica Y, Quintana R, Radeloff VC, Gavier-Pizarro G. Wetland loss due to land use change in the Lower Paraná River Delta, Argentina. Sci Total Environ. 2016; 568: 967–978. 10.1016/j.scitotenv.2016.04.200 27369090

[96] Rincón-Rubiano DR. Environmental Law in Colombia. 2nd ed Alphen aan den Rijn: Kluwer Law International; 2011.

