# Supplementary figures and images for: Wetlands are keystone habitats for jaguars in an intercontinental biodiversity hotspot
Source: PLoS One. 2019 Sep 11;14(9):e0221705. doi: 10.1371/journal.pone.0221705 (PMC6738587; doi:10.1371/journal.pone.0221705)

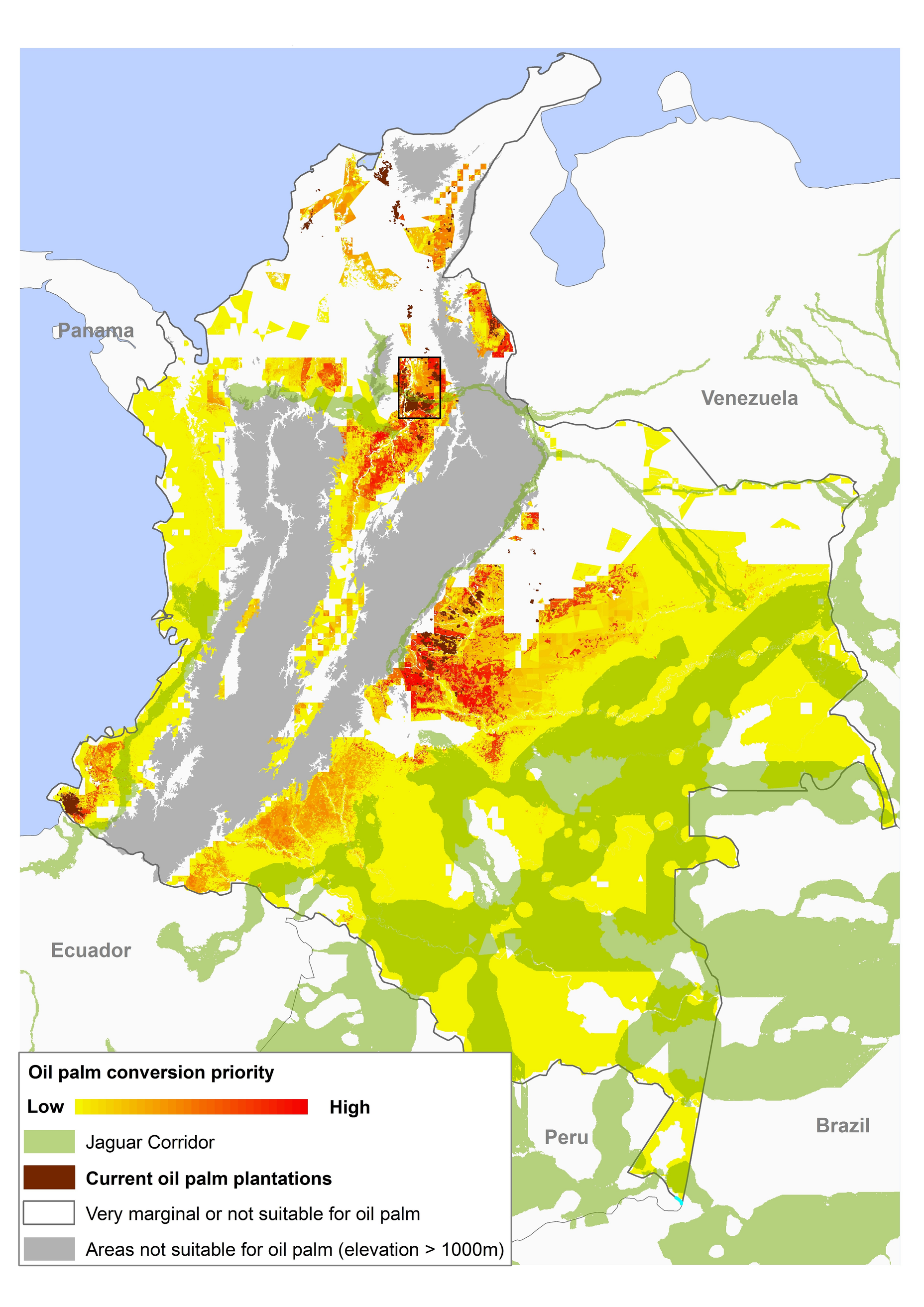

Supplement: S1 Fig — (TIF) [file pone.0221705.s001.tif]

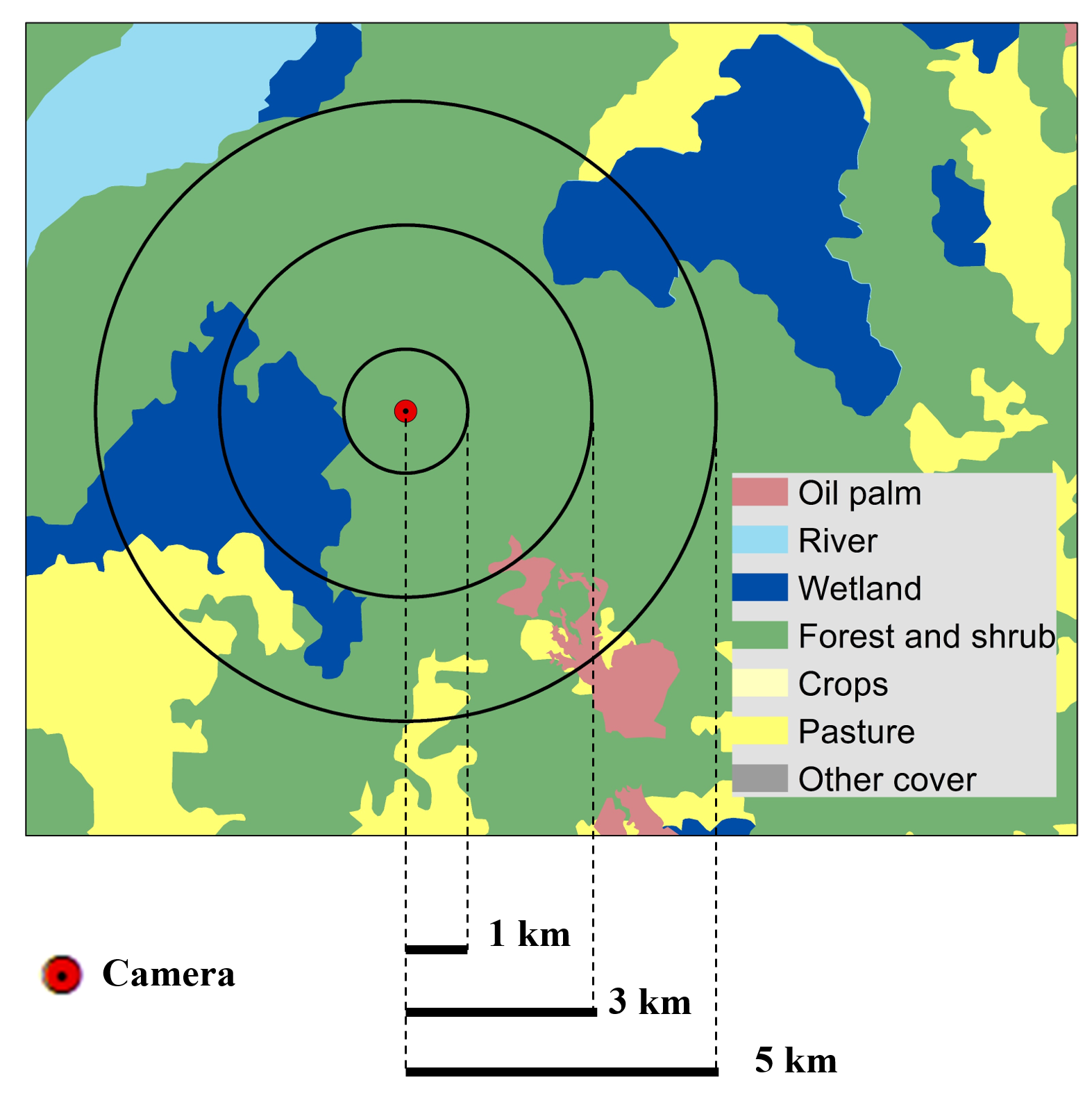

Supplement: S2 Fig — (TIF) [file pone.0221705.s002.tif]
